# Study of Natural and Artificial Aging on AlSi9Cu3 Alloy at Different Ratios of Returnable Material in the Batch

**DOI:** 10.3390/ma13204538

**Published:** 2020-10-13

**Authors:** Dana Bolibruchová, Marek Matejka, Alena Michalcová, Justyna Kasińska

**Affiliations:** 1 Faculty of Mechanical Engineering, Department of Technological Engineering, University of Zilina, Univerzitná 8215/1, 010 26 Žilina, Slovakia; danka.bolibruchova@fstroj.uniza.sk; 2 Department of Metals and Corrosion Engineering, University of Chemistry and Technology in Prague, Technická5, 166 28 Prague 6, Czech Republic; michalca@vscht.cz; 3 Department of Metal Science and Materials Technology, Kielce University of Technology, Al. Tysiąclecia Państwa Polskiego 7, 25 314 Kielce, Poland; kasinska@tu.kielce.pl

**Keywords:** Al-Si-Cu secondary aluminum alloy, returnable material, natural and artificial aging, Cu precipitate, transmission electron microscopy, mechanical properties

## Abstract

Aluminum alloys currently play an important role in the production of castings in various industries, where important requirements include low component weight, reduction of the environmental impact and, above all, reduction of production costs of castings. One way to achieve these goals is to use recycled aluminum alloys. The effect of natural and artificial aging of AlSi9Cu3 alloy with different ratios of returnable material in the batch was evaluated by a combination of optical, scanning, transmission microscope and mechanical tests. An increase in the returnable material in the batch above 70% resulted in failure to achieve the minimum value required by the standard for tensile strength and ductility. The application of artificial aging had a positive effect on the microstructure and thus on the mechanical properties of experimental alloys. By analyzing the results from TEM, it can be stated that in the given cases there is a reduced efficiency of θ’-Al_2_Cu precipitate formation with an increase of the returnable material in the batch and in comparison with artificial aging, which is manifested by low mechanical properties.

## 1. Introduction

At present, more than half of the aluminum castings produced come from recycled materials. A substantial part of the use (approximately 70%) of recycled aluminum is in the production of aluminum alloys cast mainly for the automotive industry. Secondary aluminum metallurgy as well as many other recycling processes are all important issues from both an economic and environmental point of view. The recycling process enables the economy of raw materials and energy savings [1,2,3]. Another positive aspect of aluminum recycling is the impact on the environment. The production of secondary aluminum alloys releases only 5% of greenhouse gases compared to the production of primary aluminum, as the processes associated with mining, ore refining and smelting are eliminated. For this reason, today foundries use in the batch an increasing amount of remelted material, which can make up several tens of percent of the total batch [4,5].

Despite the knowledge gained so far about the effect of multiple remelted material, there is no clear opinion on its effect. At present, determining the correct amount of remelted material in the batch is a major problem for foundries. The use of remelted return material carries with itself a high risk of a decrease in the overall quality of the casting, even though the recycled material used meets all the requirements for the input material. For this reason, it is very important to understand the remelting process and the subsequent use of the remelted material in the batch. The main goal of the paper is a more detailed description of the method and the remelted material influence in the batch on the structure and mechanical properties after natural and artificial aging. Determining the correct ratio with respect to the amount of remelted material in the batch is currently one of the key factors in increasing the competitiveness of foundries. Despite the fact that this is an important problem, it is not given sufficient attention from a scientific point of view and there are very few professional publications on the issue. The results of the experiments present how the quality of castings decreases with increasing amount of remelted material in the batch.

Al-Si-Cu alloys are the most widely used type of aluminum alloys. They make up about half of the total production of aluminum castings and are mainly used in applications for the automotive and aerospace industries. These are most often sub-eutectic (exceptionally eutectic) alloys with a content of 6 to 13 wt.% Si and 1 to 5 wt.% Cu [6]. Thanks to copper, the alloy has good mechanical properties and excellent machinability, but copper reduces the resistance to corrosion. However, this is sufficient for use in the automotive industry. Copper also allows heat treatment by hardening to form the intermetallic Al_2_Cu compound. The alloy has a low tendency to crack and shrink. To achieve these properties, it is advantageous to keep the silicon content in the upper tolerance range for the alloy [7].

Due to the increasing use of recycled aluminum alloys for demanding castings, their quality is considered a key factor and it is therefore necessary to find the right compromise between price and final quality. The quality of an aluminum alloy made from recycled materials can be affected mainly by a change in the morphology of the individual structural components. The change in morphology is most often caused by a change in the chemical composition or by the effect of multiple remelting of the alloy [8,9,10].

Different methods are used to neutralize the negative effects of remelting or an increase in the returnable material in the batch. One of the possible methods for Al-Si-Cu-based alloys is the application of precipitation hardening—aging. This is a diffusion process that takes place in the structure of the casting (often after solution annealing), in which the solid solution is depleted by additive elements. The supersaturated solid solution contains a larger amount of an additive dissolved than is thermodynamically advantageous for a given alloy at a given temperature. Such a solid solution has a natural tendency to reach the state with the lowest free enthalpy, with the following disintegration. This decay most often takes place by a heterogeneous phase transformation, i.e., precipitation [11,12,13,14].

The mechanical and physical properties of the casting change during precipitation from the supersaturated solid solution. Sensitive structural properties such as hardness, ductility, yield strength or corrosion resistance are highly dependent on the distribution of the individual phases in the structure. The greatest hardening is caused by the initial stages of precipitation. The values of yield strength, tensile strength and hardness are reduced by reaching the critical size of the precipitates in the precipitation-strengthened alloys. Precipitation can also affect the properties of the alloy in an undesirable way, such as the formation of structural instability or tempering brittleness.

The precipitation process begins with the diffusion of the additive element into microscopic regions richer in the given element, and a new phase is nucleated in them. The growth of these nuclei produces coherent precipitates referred to as Guinier–Preston zones (GPZ). Coherence means that these regions are part of the solid crystal lattice, thereby deforming the lattice and causing internal stresses in it. The induced stresses are the cause of the increasing strength and hardness of the alloy. The second stage is the formation of particles of non-equilibrium (GP II) coherent precipitates θ”. The deformation fields present in the lattice surrounding these coherent particles inhibit and prevent the movement of dislocations, thus leading to the most significant increase in strength. In the further process, the phase begins to form separate units with a gradual loss of coherence. The formations cease to be crystallographically connected to the original supersaturated solid solution, and generally partially coherent acicular-shaped particles with their own crystal lattice are formed. Such a transition precipitate—phase θ’, gradually changes size and morphology into acicular shapes.

**Figure 1:**
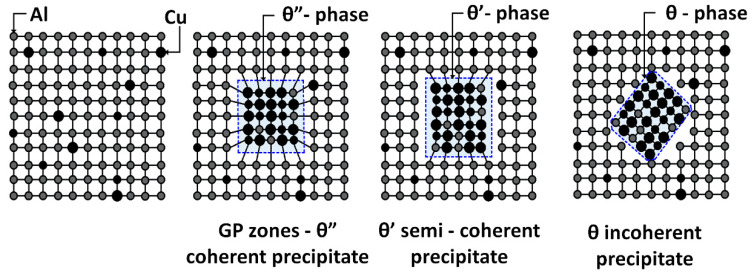
Schematic representation of the precipitation process [7].

The final stage of the aging process is the gradual loss of coherence and the formation of an incoherent particle of plate-like or lenticular shape. The incoherent hardening phase no longer has a crystalline bond to the α (Al) solid solution. At this stage, the strength and hardness decrease and the alloy becomes over-aged. A schematic representation of the precipitation process is shown in Figure 1. Maximum strength characteristics after hardening are obtained in the region of coherent precipitates θ” and at the beginning of the precipitation of the partially coherent phase θ’ [15,16,17,18,19,20].

## 2. Materials and Methods

### 2.1. Experimental Material

A sub-eutectic alloy AlSi9Cu3 (EN AC-46,000, A226) was chosen to evaluate the effect of increasing the ratio of returnable material in the batch. The primary AlSi9Cu3 alloy is characterized by medium mechanical characteristics, good strength at elevated temperatures, good foundry properties and corrosion resistance. Due to its properties, it is mainly used in automotive industry products, such as in cylinder heads and engine blocks, crankshaft housings and other components [6,7,8].

Two types of AlSi9Cu3 alloy were used in the experiment. The first type was a commercial purity AlSi9Cu3 secondary alloy ingots purchased from the company Dor, Považská Bystrica, Slovakia. The other type of AlSi9Cu3 alloy was prepared by remelting a typical foundry returnable material, such as ingot remnants, gating and riser systems. The foundry returnable material was remelted and cast into the shape of ingots. The remelting of the returnable material with a total batch weight of 95 kg took place in an electric resistance furnace with a volume of 100 kg in a steel crucible with applied protective graphite coating. The chemical composition of the alloy according to the standard (EN 1706) of the secondary alloy (commercial purity) and the alloy prepared by remelting the foundry returnable material is given in Table 1.

### 2.2. Experimental Methods

In the next part of the experiment, five alloys were cast in succession with the designation 20–80; 50–50; 70–30; 80–20; and 90–10, where the first number indicates the percentage of returnable material and the second number the proportion of secondary alloy in the batch. Each batch weighed 12.5 kg. Melting was performed in an electric resistance furnace (LAC, Židlochovice, Czech Republic) with a T15 type regulator with a capacity of 15 kg in a graphite crucible, which was treated with a protective coating. The casting took place at a temperature of 750 ± 5 °C. The samples were cast from each melt for structural analysis and mechanical property tests (10 samples) under the same conditions. Casting was performed into a metal mold with a temperature of 150 ± 5 °C. We did not refine, inoculate or modify the alloys in any way in the process, and only the oxide membranes were mechanically removed before casting. The chemical composition of the newly formed alloys is given in Table 2. Structural analysis and mechanical characteristics of the samples were evaluated after natural aging (NA—approximately 160 h at 20 °C) and after artificial aging T5 (AA—at 200 ± 5 °C for 4 h and cooling in water with a temperature of 60 ± 5 °C). Chemical composition was measured using arc spark spectroscopy (Bunker-Q2 ION, Kalkar, Germany).

Samples (10 mm × 10 mm) for metallographic evaluation were prepared by standard metallographic procedures (coarse and fine wet grinding, polishing on an automatic instrument using a diamond emulsion, and etching). For light microscopy purposes, samples were etched with 20 mL H_2_SO_4_ + 100 mL distilled water to enhance the ferrous intermetallic phases.

For SEM (scanning electron microscope) and EDX (energy dispersive X-ray) analysis, samples were etched with 0.5% HF solution. For deep etchings, an etchant consisting of 36 mL HCl + 100 mL H_2_O was used, etching the surface of the samples for approximately 30 s, to reveal the three-dimensional morphology of eutectic silicon and intermetallic phases. (α-phase) lattice residues were removed by vigorous rinsing with alcohol-based liquid. The microstructure of the experimental material was evaluated using a NEOPHOT 32 optical microscope and SEM observations with EDX analysis using a VEGA LMU II scanning electron microscope (Tescan, Brno, Czech Republic) connected to energy dispersive X-ray spectroscopy (BruckerQuantax EDX analyzer, Bunker, Kalkar, Germany).

Observation of the structure by TEM (transmission electron microscopy) and by the experimental technique of SAED (Selected Area Electron Diffraction) was performed on a Jeol 2200 FS device (JEOL Ltd., Tokyo, Japan). Samples were prepared by re-polishing using a Gatan PIPs (precision ion polishing system, Gatan, Pleasanton, CA, USA). The principle consists in de-dusting the sample with a stream of ionized argon. The samples were circular in shape with a diameter of 3 mm and a thickness of 70 micrometers. At the beginning of re-polishing, an angle of 8° was used, and when an orifice appeared in the sample, the angle was gradually reduced to 4° and 2° on both sides of the sample. This ensured that the area around the orifice was thin enough to allow the material to be observed. For TEM, the sample is transparent to a thickness of 100 nm. Such a procedure makes it possible to maintain more massive edges and to have the center of the sample transparent.

The evaluation of microhardness of structural components was performed on a Hanemann device type Mod 32 at a load of 10 p. The resulting value presents the average value from 20 measurements.

The tensile strength test was performed according to EN 42 0310 using a WDW 20 device (Jnkason, Jinan, China). The maximum load of the device was 20 kN at a constant crosshead feed rate of 2 mm/min. The evaluation of ductility A_50_ was performed on the MC electronic manual extensometer device (Jnkason, Jinan, China). The cast samples were mechanically machined into test bars with a diameter of 10 mm in five pieces for each condition. The Brinell method on an INNOVATEST NEXUS 3002XLM-INV1 hardness tester (Innovatest, Borgharenweg, Maastricht, The Netherlands) was used to measure hardness. A 5 mm diameter bead with a load of 125 kP (1226 N) was pressed into the prepared sample for 15 s. A minimum of five measurements were made for each sample.

Twelve castings were cast in each melt. Finished castings were removed from the mold after approximately 60 s in an effort to maintain the same solidification conditions for each casting.

## 3. Results

### 3.1. Mechanical Properties

**Figure 2:**
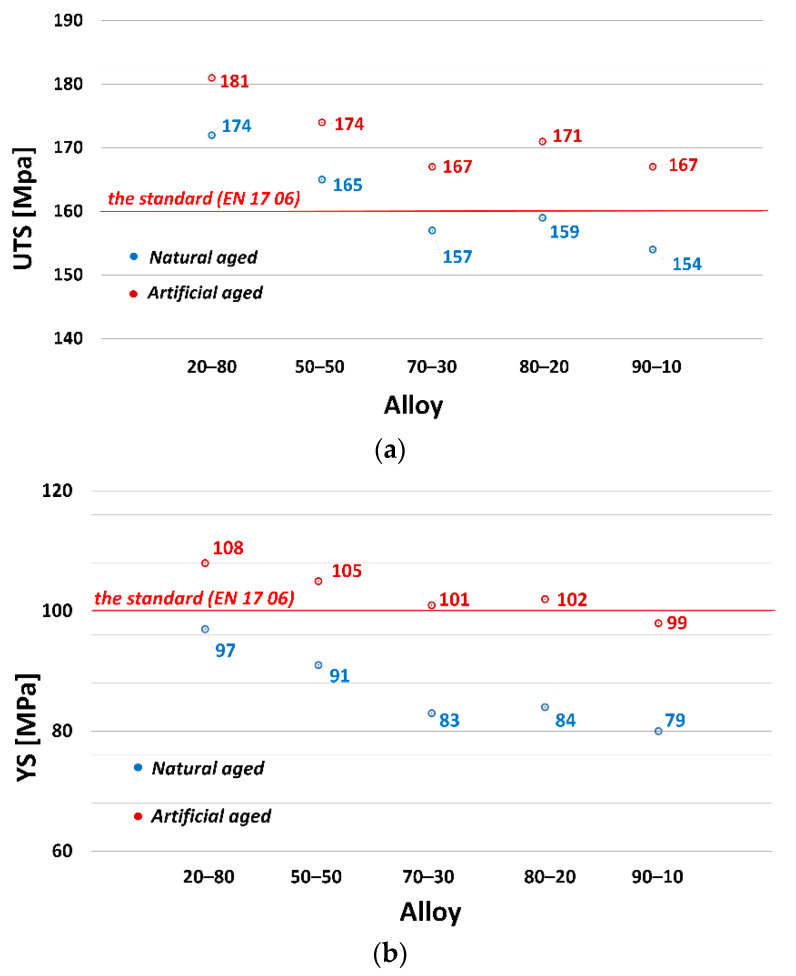
Relationship between: (**a**) Ultimate strength tensile; (**b**) Yield strength and experimental alloy and the state the alloy.

**Figure 3:**
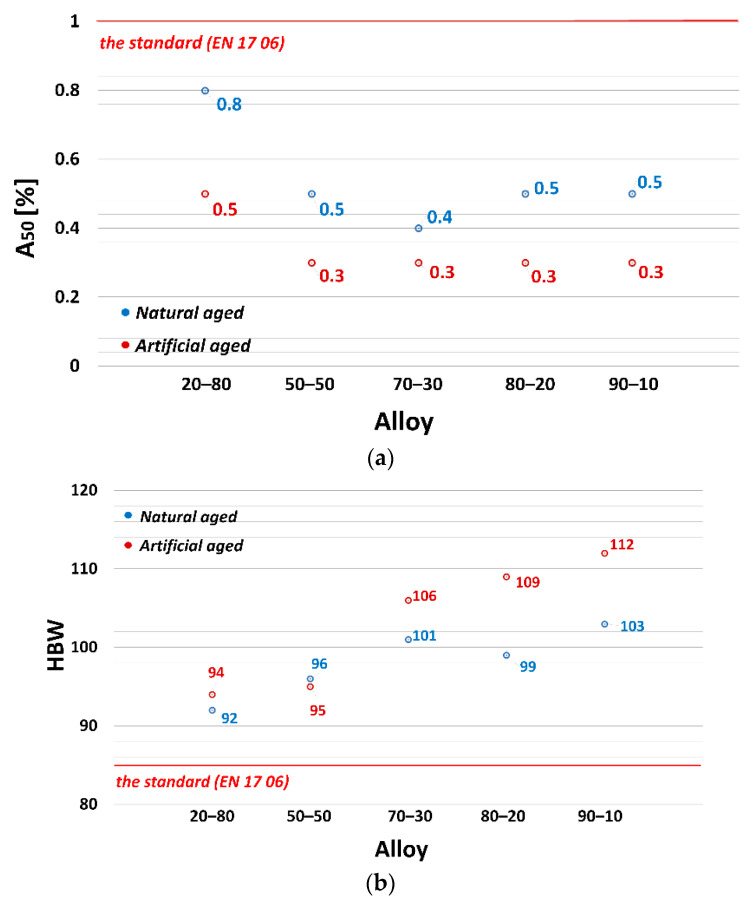
Relationship between: (**a**) Elongation; (**b**) Brinell hardness and experimental alloy and the state the alloy.

The influence of applying natural and artificial aging on AlSi9C3 alloy with different amounts of returnable material in the batch on mechanical properties (Ultimate tensile strength—UTS, Yield strength—YS, Ductility—A_50_ and Brinell hardness—HBW) is shown in Figure 2 and Figure 3. The numerical value represents the average value of the five measurements for both alloy states.

The best values were obtained for the alloy with a 20% returnable material content, UTS = 174 MPa (Figure 2a) and YS = 97 MPa (Figure 2b). By increasing the proportion of returnable material to 70%, there was a gradual decrease in the observed characteristics. From the alloys with the ratios 70–30 to 90–10 there was a stabilization and the values ranged at approximately the same levels. The lowest values of UTS = 154 MPa and YS = 79 MPa were measured for the alloy with the highest amount of returnable material—the 90–10 alloy. By applying artificial aging (T5) (red dots on the graph), an increase was achieved in tensile strength and agreed yield strength in all alloys. The resulting values of UTS and YS show a slight increase in artificial aging (200 °C/4 h). For the 20–80 alloy, the increase in UTS was approximately by 4% due to artificial aging, and for alloys with 70% and higher ratios, the increase in UTS was approximately by 8% compared to natural aging. The red line in the graphs indicates the minimum values required by the standard (EN 17 06) for gravity-cast AlSi9Cu3 alloy.

A decreasing trend was also observed in the evaluation of ductility (Figure 3a). Due to the increasing proportion of returnable material in the batch, there was a significant decrease in ductility values. From the maximum measured value of A_50_ = 0.8% (20–80 alloy) to the value of A_50_ = 0.4% (70–30 alloy). After artificial aging, there was a decrease in ductility in all cases. The minimum ductility A_50_ = 0.3% was measured for alloys with 50% and higher proportion of returnable material in the batch. The resulting HBW hardness values (Figure 3b) had an increasing trend due to the increase in the returnable material in the batch. The increase in the HBW hardness can be attributed to the increasing weight % of Fe in the alloys, which predetermines the increased number of iron phases in the structure and thus the increase in hardness [10,21]. Artificial aging, as expected, increased HBW hardness values. Maximum HBW hardness values were measured for the 90–10 alloy, namely, HBW = 103 (natural aging) and HBW = 112 (artificial aging). The red line in the graphs indicates the minimum values required by the standard (EN 17 06) for gravity cast AlSi9Cu3 alloy.

### 3.2. Microstructure

#### 3.2.1. Natural Aging

**Figure 4:**
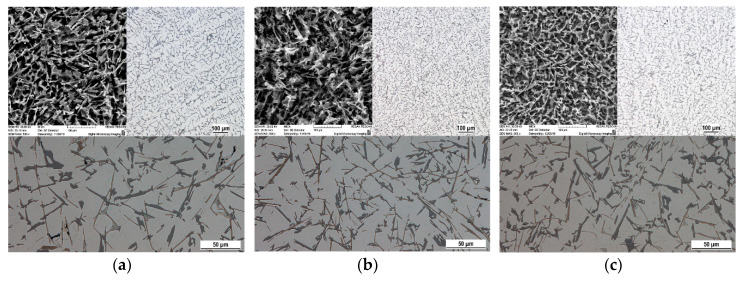
Microstructure of AlSi9Cu3 alloy depend on the ratio of returnable material in the batch after natural aging, SEM; (**a**) 20–80 alloy, (**b**) 50–50 alloy, (**c**) 90–10 alloy.

**Figure 5:**
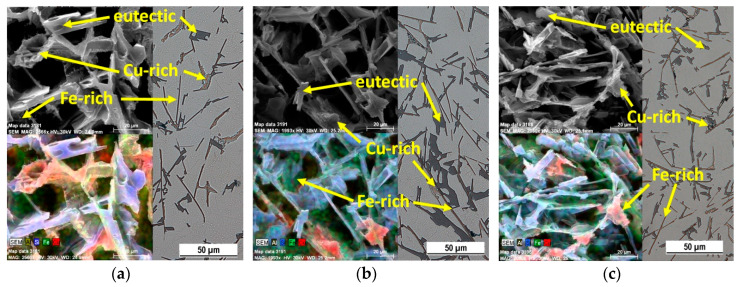
Changes of eutectic Si, Cu-rich and Fe-rich phases of AlSi9Cu3 alloy depend on the ratio of returnable material in the batch after natural aging, SEM; (**a**) 20–80 alloy, (**b**) 50–50 alloy, (**c**) 90–10 alloy.

The structure of the subeutectic AlSi9Cu3 alloy consists of α-phase (pale gray), eutectic (dark gray) and intermetallic phases with different chemical compositions, most often based on Cu and Fe. In the microstructure of the 20–80 experimental alloy, eutectic grains are present in the so-called unmodified shape as hexagonal plate crystals with unoriented distribution. Iron-based intermetallic phases crystallized in interdendritic regions, most frequently as Al_5_FeSi phases in the plate morphology (aciculars in metallographic cutting) (Figure 4a and Figure 5a). Copper-rich intermetallic phases were primarily excreted near eutectic silicon grains, especially aciculars of iron phases, mainly as Al_2_Cu in the form of Al-Al_2_Cu-Si containing about 24 wt.% Cu. The increased proportion of returnable material resulted in a local thickening of the eutectic silicon grains, an increase in the lengths of the iron phase aciculars, and in their local thickening (Figure 4b,c and Figure 5b). Cu-based intermetallic phases were also observed in alloys with 70% and higher returnable material content with increased Fe content as Al_7_FeCu_2_ (Figure 5c).

#### 3.2.2. Artificial Aging

**Figure 6:**
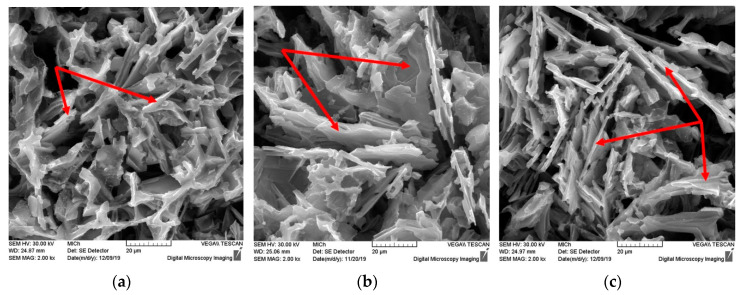
Changes of eutectic Si of AlSi9Cu3 alloy depend on the ratio of returnable material in the batch after artificial aging, SEM; (**a**) 20–80 alloy, (**b**) 50–50 alloy, (**c**) 90–10 alloy.

The application of artificial aging did not significantly change the morphology of eutectic silicon. For the 20–80 alloy, a hexagonal plate formation with well-recognizable twinning could be observed (Figure 6a). Artificial aging caused only local growth of the arched edges without subsequent thinning and fragmentation. Similarly, without heat treatment, eutectic silicon in the 50–50, 70–30 and 90–10 alloys is present in an undirected distribution of plates, or as unmodified eutectic (Figure 6b,c).

**Figure 7:**
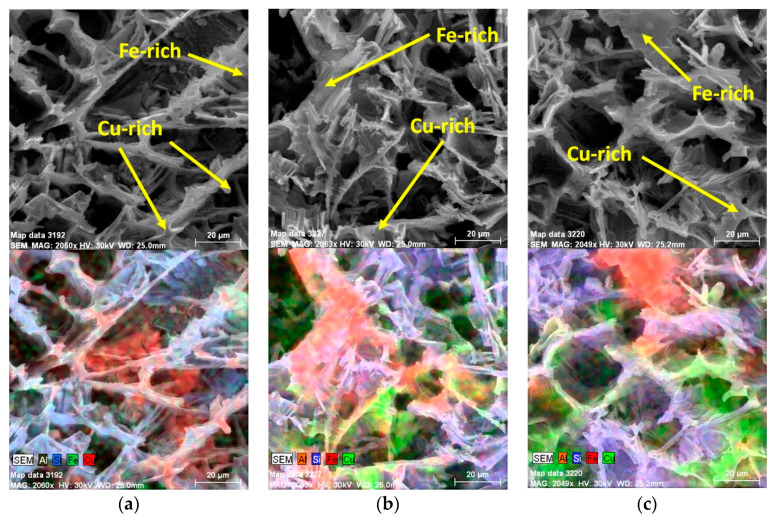
Changes of Cu-rich and Fe-rich phases of AlSi9Cu3 alloy depend on the ratio of returnable material in the batch after artificial aging, SEM; (**a**) 20–80 alloy, (**b**) 50–50 alloy, (**c**) 90–10 alloy.

Artificial aging had no significant effect on the morphology of the copper- and iron-rich phases (Figure 7). Cu-based intermetallic phases are present as Al_2_Cu with tetragonal crystal lattice and Al_7_FeCu_2_ [22,23]. In all experimental alloys, artificial aging resulted in a local shortening of Fe-based intermetallic phase lengths compared to natural aging.

### 3.3. Substructure

Two types of alloys were selected for transmission electron microscopy (TEM), with the lowest (20–80) and highest (90–10) ratios of returnable material in the batch after natural and artificial aging. Experimental alloys 20–80 and 90–10 represent alloys with the best or the worst results of mechanical tests. The use of TEM is the most effective way to observe the occurrence of the metastable phase θ’-Al_2_Cu, which is responsible for precipitation hardening in the alloy and fundamentally affects the mechanical properties. The elevated temperature precipitation follows the same mechanism for crystalline [24] and also for amorphous alloys [25]. At first, the non-stoichiometric clusters are formed due to diffusion. This local density variation serves as nuclei for precipitates growth. The precipitates follow during their growth crystallography of matrix grain [24] or even crystallography of the substrate in case of amorphous matrix [26]. The formation of precipitates can be predominantly proven by SAED because of its sufficient spatial resolution.

#### 3.3.1. 20–80 Alloy

**Figure 8:**
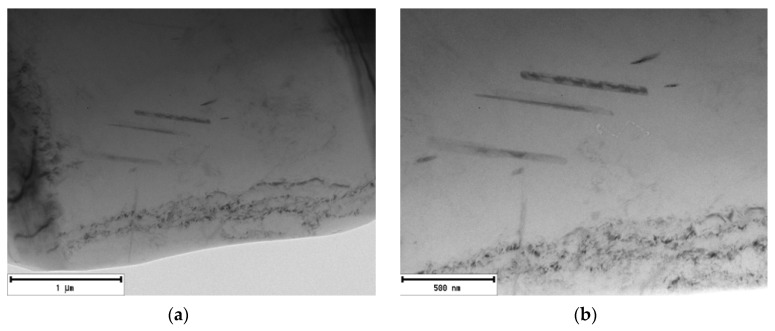
Substructure of 20–80 alloy after natural aging, TEM; (**a**) Overview (**b**) Detailed view.

In the alloy with the lowest amount of returnable material after natural aging, precipitates are present in the morphology of thicker rods with an approximate length of 800 nm (Figure 8). The presence of a structural defect is indicated at the bottom of the image (dark spots). These are mainly clusters of dislocations, or small-angular grain boundaries, but the shown defects could also be introduced by the sample preparation process (mechanical grinding, ion polishing).

**Figure 9:**
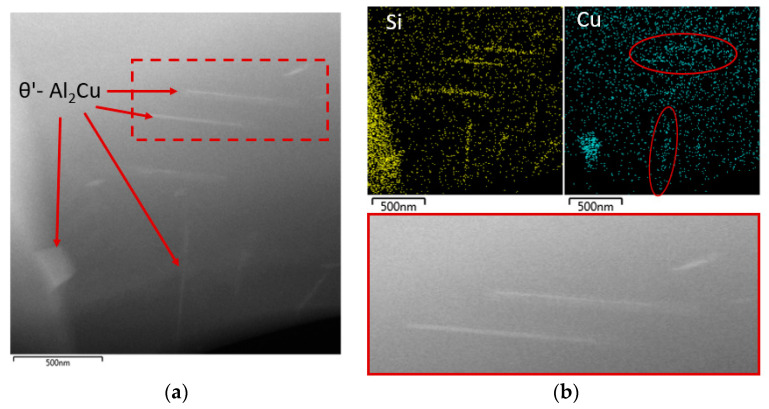
Substructure of 20–80 alloy after natural aging focus on θ’-Al_2_Cu phases, TEM, STEM/HAADF; (**a**) 20–80 alloy, (**b**) Morphology of the θ’-Al_2_Cu phase and distribution of Si and Cu elements and detail of needle morphology of θ’-Al_2_Cu phase.

**Figure 10:**
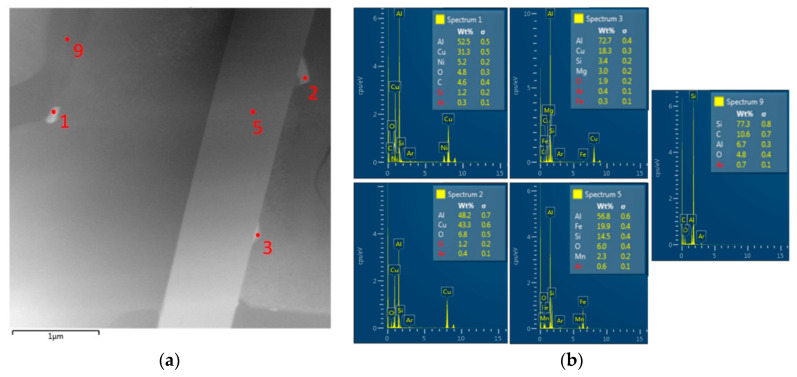
Point EDX analysis of 20–80 alloy substructure after natural aging; (**a**) Places of point EDX analysis, (**b**) Results of chemical composition of structural components.

The application of EDX mapping showed that the rod particles are primarily formed by Si (Figure 9). In more detail, and using the HAADF (high annular angular dark field) mode, the presence of the θ’-Al_2_Cu phase was shown in the acicular morphology located in close proximity to the Si particles (Figure 9b). In addition to the acicular morphology, the θ’-Al_2_Cu phase is also in the morphology of the sharp-edged plate formation (Figure 9a). The sharp-edged θ’-Al_2_Cu phase was also observed in the subsequent evaluation, especially near the Si- and Fe-based phases. Spot EDX analysis showed an increased presence of Cu in phases designated 1, 2 and 3 (Figure 10).

**Figure 11:**
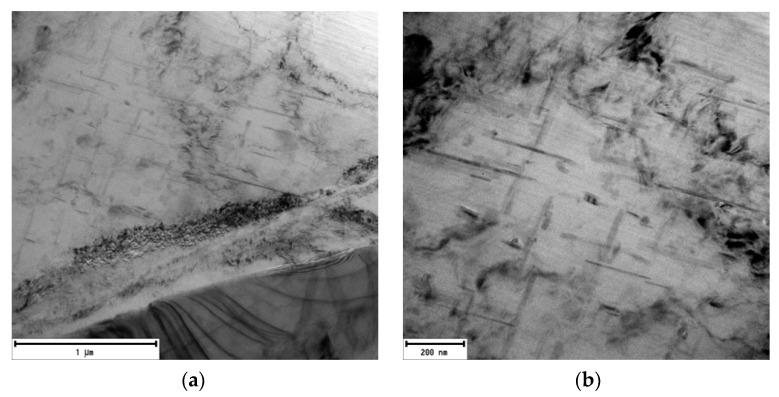
Substructure of 20–80 alloy after artificial aging, TEM; (**a**) Overview (**b**) Detailed view.

**Figure 12:**
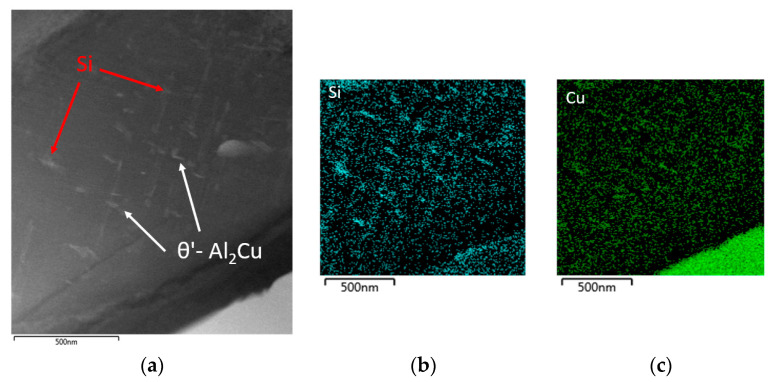
Substructure of 20–80 alloy after artificial aging TEM; (**a**) θ’-Al_2_Cu and Si phases, (**b**) mapping of Si (**c**) mapping of Cu.

The substructure of the 20–80 alloy after artificial aging, which exhibited “ideal” properties, is characterized by a large number of perpendicularly oriented acicular particles. It is a rapid increase in these particles compared to the alloy after natural. Structural disorders (especially clusters of dislocations) are present to approximately the same extent as in natural aging (Figure 11). Similar to the 20–80 alloy, after natural aging, perpendicularly oriented acicular particles are exhibited by θ’-Al_2_Cu phases and Si-rich phases. During the application of mapping, phases were observed again in acicular and also plate morphology (Figure 12).

**Figure 13:**
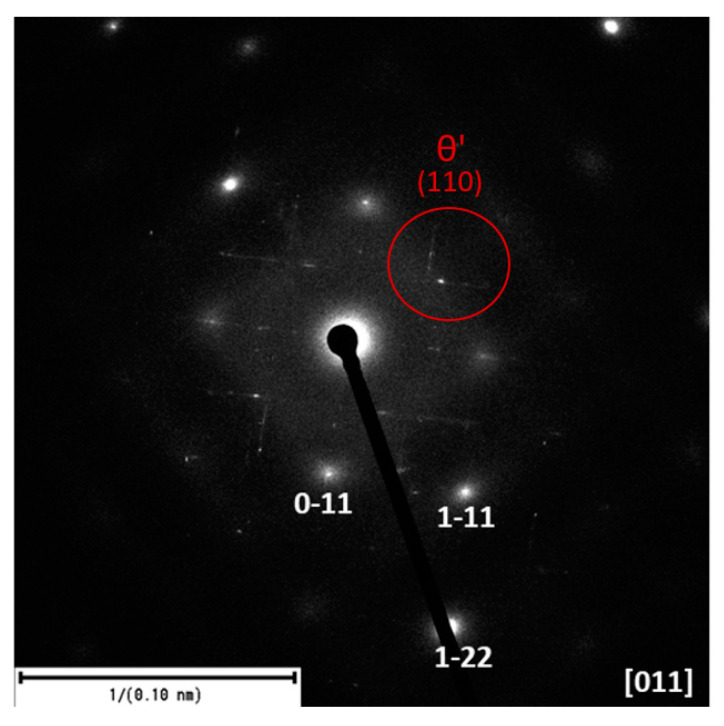
SAED of θ’-Al_2_Cu phases of 20–80 alloy after artificial aging.

The experimental technique of SAED (selected area electron diffraction) forming a part of the transmission electron microscope was used to evaluate the substructure of the 20–80 alloy after artificial aging. SAED provides information on crystallography and orientation of crystalline materials (Figure 13). The regularity of the maxima (dashes) of precipitates θ’ can be observed in the SAED image, which indicates their regular orientation towards the matrix and is exactly crystallographically given.

#### 3.3.2. 90–10 Alloy

**Figure 14:**
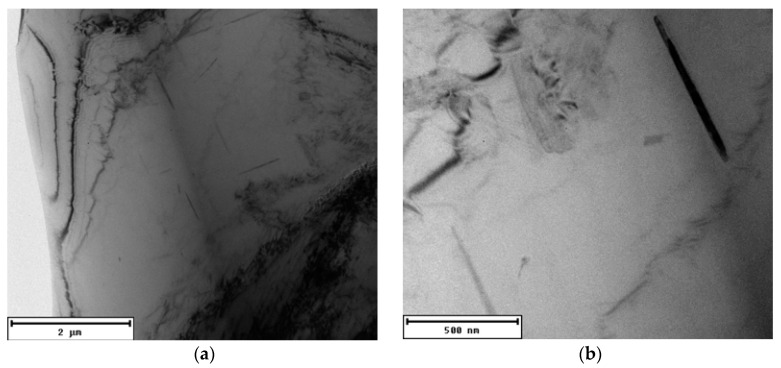
Substructure of 90–10 alloy after natural aging, TEM; (**a**) Overview (**b**) Detailed view.

**Figure 15:**
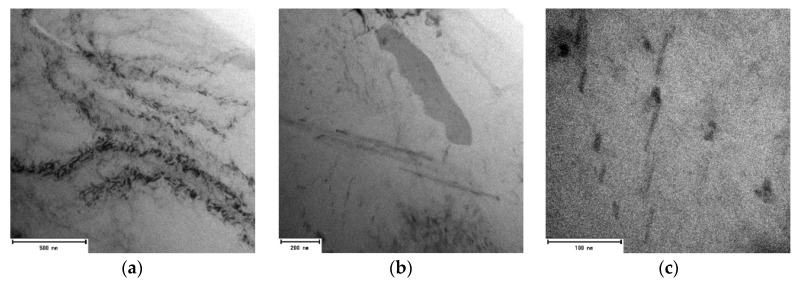
Substructure of 90–10 alloy after artificial aging, TEM; (**a**) Overview (**b**) Detailed view (**c**) High resolution.

In the substructure of the alloy with the highest amount of returnable material after natural aging, similarly to the 80–20 alloy, the presence of a smaller number of rod formations with a length of 500 to 1000 nm and lattice defects of the dark cluster can be seen (Figure 14). Application of artificial aging in the 90–10 alloy allows us to observe increased occurrence of particles in acicular morphology, but also in plate-like one with rounded edges (Figure 15).

**Figure 16:**
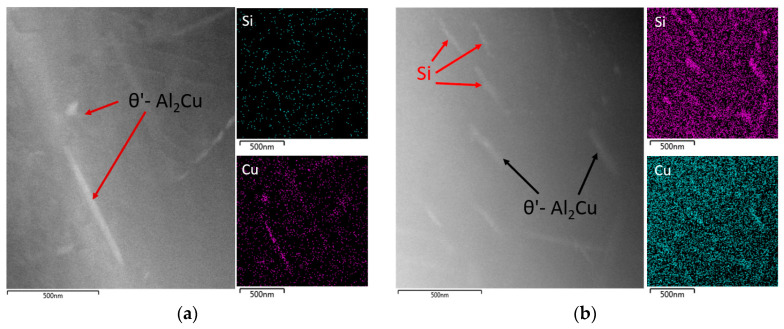
Substructure of 90–10 alloy focus on θ’-Al_2_Cu phases, TEM; (**a**) natural aging, (**b**) artificial aging.

Element mapping confirmed the presence of θ’-Al_2_Cu precipitates in both alloys (both after natural and artificial aging) (Figure 16). In the 90–10 alloy after artificial aging, Si-based particles were also present in the substructure along with the precipitates. (Figure 16b).

**Figure 17:**
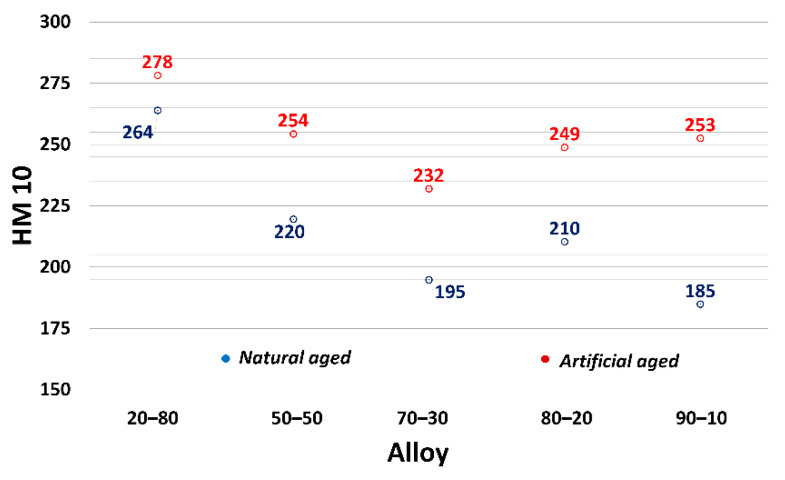
Dependence of the microhardness of the primary α-phase on AlSi9Cu3 alloys from the different ratio of remelted returnable material in the batch.

The microhardness evaluation of the primary α-phase confirmed the increased presence of the θ’-Al_2_Cu phase after the application of artificial aging that is responsible for precipitation hardening in AlSi9Cu3 alloys. The trend of the microhardness values was decreasing, with increasing ratio of returnable material in the batch (with the exception of the 80–20 alloy) (Figure 17). The application of artificial aging resulted in increased values of all alloys, which confirms the increased ability to form precipitates in the substructure of experimental alloys.

## 4. Discussion

The evaluation of the obtained results confirmed the expected decrease in the overall quality of the AlSi9Cu3 alloy due to the increasing proportion of recycled material in the batch from 20% to 90%. The negative effect of the recycled material increase in the batch was already manifested at the amount of 50% (alloy 50–50). The microstructure of alloy 50–50 is characterized by an increased presence of harmful iron phases and by the degraded grains of Si compared to an alloy with a lower amount of recycled material in the batch (alloy 20–80). By further increasing the amount of recycled material in the batch to the level of 70% to 90%, a large amount of harmful iron phases was formed, which had a major impact on the gradual decrease of the investigated mechanical characteristics except for the hardness of these alloys (70–30, 80–20 and 90–10) [27,28,29]. Compared to the EN 17 06 standard, only the alloys with a high proportion of returnable material (70–30, 80–20, 90–10) did not reach the required minimum tensile strength, and the minimum agreed yield strength was not reached by any investigated alloy after natural aging.

The application of artificial aging had a positive effect on the length of the acicular iron phases and local refinement of eutectic silicon, and thus on the resulting mechanical properties of the investigated alloys, which in all cases exceeded the minimum value required by the standard when assessing tensile strength and agreed yield strength. On the contrary, it had a negative effect only when evaluating the ductility of alloys with different amounts of returnable material in the batch.

The use of transmission electron microscopy confirmed the decreasing ability of the AlSi9Cu3 alloy to age naturally with an increase in the returnable material in the batch. The substructure of the alloy with 90% returnable material (the 90–10 alloy) is characterized by only a very small number of coherent and semi-coherent θ’-Al_2_Cu phases compared to the substructures of the 20–80 alloy. The reduced presence of curable Al_2_Cu phases was also confirmed in the microhardness measurement, by decreasing the values of the primary α-phase with a gradual increase in the returnable material content in the batch.

By applying artificial aging (200 °C/4 h), a rapid increase of θ’-Al_2_Cu phases in acicular morphology was observed in the substructure of the 20–80 alloy. The increased ability to form coherent and semi-coherent θ’-Al_2_Cu phases using artificial aging was also shown in the alloy with the highest amount of returnable material (the 90–10 alloy).

## 5. Conclusions

The aim of this study is to describe the change in the effectiveness of natural and artificial aging on the AlSi9Cu3 alloy with an increasing proportion of returnable material in the batch. The results of the study confirm that the morphology of the eutectic silicon degrades and the lengths of the iron phases increase due to the increase in the returnable material in the batch. The results also show a decrease in the ability to form θ’-Al_2_Cu precipitates in the structure of alloys with 50% and higher proportions of returnable material in the batch. This suppressed the ability of the self-hardening process that is characteristic of AlSi9Cu3 alloy. Degradation of morphology and suppression of the self-hardening process were accompanied by a decrease in tensile strength, agreed yield strength and ductility. The above results show that although the use of returnable material in the batch as a substitute for primary alloys has a significant economic and environmental aspect in foundry production, a negative decrease in mechanical properties of experimental alloys due to reduced efficiency of θ’-Al_2_Cu precipitate formation was confirmed.

The application of artificial aging had a positive effect on the overall quality of experimental alloy castings, thus expanding the possibilities of using castings created mostly by recycled material. Artificial aging had a positive effect on the microstructure and substructure, which was subsequently reflected in the improvement of the mechanical properties of the castings. Increased efficiency of artificial aging compared to natural aging was manifested especially in alloys with 50% and higher proportion of returnable material in the batch (which gradually lost the ability to self-cure). Artificial aging led to increased diffusion of the additive element into microscopic areas and thus to nucleation of a new phase, which restored the alloys’ ability to form precipitates in the substructure.

## Figures and Tables

**Table 1:** Chemical composition of primary AlSi9Cu3 by standard, secondary alloy and returnable materials of AlSi9Cu3 alloy (wt.%).

Elements	Si	Fe	Cu	Mn	Mg	Ni	Zn	Ti	Cr
Primary AlSi9Cu3 (EN 1706)	8.0–11.0	0.6–1.1	2.0–4.0	0.55	0.15–0.55	0.55	1.20	0.20	0.15
Commercial purity AlSi9Cu3	9.563	1.081	2.206	0.184	0.426	0.092	1.160	0.038	0.027
Returnable AlSi9Cu3	9. 294	1.674	2.074	0.184	0.348	0.129	1.016	0.034	0.113

**Table 2:** Chemical composition of experimental alloys AlSi9Cu3 alloy (wt.%).

Elements	Si	Fe	Cu	Mn	Mg	Ni	Zn	Ti	Cr
20–80	9.507	1.294	2.197	0.231	0.391	0.122	1.044	0.035	0.049
50–50	9.418	1.419	2.173	0.223	0.361	0.134	1.041	0.033	0.072
70–30	9.245	1.569	2.02	0.209	0.344	0.108	0.961	0.031	0.112
80–20	9.415	1.617	2.08	0.206	0.358	0.156	1.07	0.032	0.101
90–10	9.291	1.643	2.143	0.199	0.357	0.127	1.046	0.032	0.106
